# Extracellular Vesicles and Interleukins: Novel Frontiers in Diagnostic and Therapeutic for Cancer

**DOI:** 10.3389/fimmu.2022.836922

**Published:** 2022-03-21

**Authors:** Aline G. Souza, Leandro M. Colli

**Affiliations:** Department of Medical Imaging, Hematology, and Oncology, Ribeirao Preto Medical School, University of Sao Paulo, Ribeirao Preto, Brazil

**Keywords:** biomarkers, personalized medicine, cytokine, targeted therapies, cancer

## Abstract

Tumor cells present many strategies for survival and dissemination in the tumor environment. Extracellular vesicles are a vital pathway used in crosstalk between tumor and non-malignant cells. They carry different types of molecules that, when internalized by target cells, can activate signaling pathways and molecular processes that will promote and disseminate neoplastic cells. Proteins, nucleic acids, and different cytokines, such as interleukins, are the main classes of molecules carried by extracellular vesicles and are being studied to understand the molecular mechanisms present in the tumor microenvironment. In particular, although poorly understood, the association between EVs and interleukins has revealed potential approaches to the diagnosis and therapeutics of several neoplasms.

## Introduction

Cell-cell communication is an event coordinated and critical in all biological processes, such as proliferation, differentiation, and apoptosis. Thus, abnormal cell-cell interaction can result in tumorigenic events (1). The primary forms of communication between cells include cell-to-cell contact signaling and the secretion of several soluble regulatory factors such as hormones, growth factors, cytokines, and chemokines. Recently, extracellular vesicles (EVs) have been considered the third mechanism of intercellular communication (2–6). EVs are a heterogeneous groups of membrane-bounded vesicles containing different molecular components, such as nucleic acids, proteins, lipids, and metabolites, which can be internalized by recipient cells, triggering biological changes (3, 5, 7).

The biogenesis of EVs can occur in two main ways: released by external budding from the cell’s plasma membrane, and fusion of multivesicular bodies of endosomal origin with the plasma membrane. EVs released by cells vary in size and are classified based on the process of biogenesis as: apoptotic bodies, exosomes, ectosomes, microvesicles and large oncossomes (8–14). Apoptotic bodies are vesicles usually released during programmed cell death and have a size between 50 to 5000 nm in diameter (15, 16). This vesicle can carry spliceosomal proteins and promote alterations in RNA splicing in recipient cells. For instance, in glioblastoma, the exogenous RBM11 protein present in apoptotic vesicles is able to change the splicing of the *MDM4* gene and *cyclin D1* to oncogenic isoforms (17). Exosomes, despite having a smaller size, between 40 to 160 nm in diameter, can also transfer molecules with the ability to stimulate tumorigenesis. *In vitro* studies with cancer cell lines, and *in vivo* analyzes with biological fluids show a significant increase in the release of exosomes by neoplastic cells (18, 19). Furthermore, tumor cell-derived exosomes are involved in many biological events related to the development and promotion of cancer, such as tumorigenesis, mesenchymal epithelial transition, drug resistance and metastasis (20–23). Ectosomes, as well as microvesicles are formed by direct budding of the plasma membrane. These vesicles are larger than exosomes (250–1000 nm) and can be released by different cells, including tumor cells (6, 12). Finally, studies have demonstrated the existence of vesicles derived from tumor cells with an atypical size between 1-10 μm in diameter, called large oncosome, directly associated with cancer progression (13, 24). Despite the different existing classifications for the set of EVs released by cells, there is still no consensus on the nomenclature of the extracellular vesicles secreted by cancer cells. Thus, according to the guidelines of the International Society of Extracellular Vesicles (ISEV) to avoid misinterpretation, herein, we adopt the term ‘EVs’ to address any vesicle subtype (25).

Almost all cells can secrete different types of EVs; however, the cargo of each vesicle will depend on the cell type and physiological state that cell finds itself during EVs biogenesis (26, 27). Tumors present a constant communication between their cells, and EVs may contribute to this intercellular crosstalk by establishing a favorable microenvironment for malignant transformation since the content present in each EV can reprogram events such as angiogenesis and modulate the immune. However, the molecular signatures of EVs derived from the tumor vary according to the type and degree of aggressiveness (28–31).

In personalized medicine, EVs have been approached as tools for performing liquid biopsy applied to detect diseases such as cancer and monitoring of therapeutic responses. Therefore, EVs have being considered potential biomarkers in cancer (32, 33). On the other hand, the EVs produced by cancer cells or immune cells may drive the regulation of immunity by transducing different signals, contributing, for example, to cytokine production and consequently favoring tumor growth, metastasis, and immune activation and suppression during the tumorigenesis (28, 34). Targeting those EV effects could open opportunities for new treatments.

Cytokines are proteins that regulate the growth and differentiation of immune cells and the activation of the immune system. They are commonly released in response to cellular stress, such as in situations of carcinogen injury, to control inflammatory or infectious processes (35). However, there are cases where there is continuous production of these molecules, characterizing a picture of chronic inflammation, impacting several processes that favor the tumor microenvironment. Among the cytokine family, several interleukins can be considered essential for cancer development and progression since activation of some interleukins after the oncogenic event can become a pathological mechanism that contributes to tumor growth and metastatic dissemination (28, 36, 37). The interleukins are a family of several molecules (IL-1 to IL-36), synthesized mainly through leukocytes. Like other cytokines, the mechanism of action in cancer is specific for each interleukin that activates a subset of cells with the corresponding receptor (37).

In this review, we highlight the ability of the content present in EVs to regulate important cytokines and present association studies between EVs and cytokines used in cancer investigations and therapeutic approaches.

## The Role of EVs in Delivering Interleukins

Most secreted proteins, including cytokines, carry an N-terminal signal peptide and/or a transmembrane domain that directs these proteins to their extracellular destination. However, cytokines and chemokines can reach the extracellular space being packaged in EVs, thus facilitating the delivery and targeting of these molecules to distant cells (38). Furthermore, EVs can be targeted to target cells through the binding of surface cytokines of EVs to cells that express specific cytokine receptors (39). Likewise, MHC I and II receptors, transferrin receptors and tetraspanins present in the membrane structure of EVs can promote their targeting and the engineering of EVs for therapeutic purposes (40, 41). In this regard, it is possible to consider the technological advances of engineering EVs to provide therapeutic tools for the treatment of diseases such as cancer. For instance, in lung cancer, 3LL tumor cells were genetically modified to release exosomes with CD40L overexpression and high levels of interleukin-12, promoting the maturation of dendritic cells, and consequently the increase in T cell proliferation and antitumor activity *in vivo* (39). However, cytokine packaging by EVs is also present in cells in their normal state. Recently, Fitzgerald et al. showed that the cytokine secretion process from tonsillar tissue, placental villous explants, whole blood, amniotic fluid and, platelet poor plasma are heterogeneous, varying between free and EV-encapsulated forms and that the proportion of cytokines encapsulated with EVs is dependent on its origin. Among the 33 cytokines evaluated, ten interleukins (IL-2, IL-4, IL-10, IL-12, IL-15, IL-16, IL-18, IL-21, IL-22, IL-33) were more frequently associated in the surface or internal to EVs (42). This association contributes to the delivery and targeting of these molecules to distant target cells since delivery is mediated by cytokine receptors present in target cells. Also, EVs may improve the production of interleukins and chemokines by stimulating cell resistance under cytotoxic stress conditions to facilitate tumor progression and protect cytokines from degradation by encapsulation (42, 43).

### EVs Cargo Controls Interleukins Function

To achieve efficient communication, EVs can interact with receptor cells through different pathways, including the release of the EVs cargo in the extracellular environment and interaction with cell membrane receptors in the process of fusion or endocytosis. The possibility of interaction with cell receptors allows EVs to address the content of these vesicles to specific cells, generating different biological responses in the recipient cell (44–47). For instance, in the immunological scenario, the alteration of different cytokines can be driven by molecules present in tumor-derived EVs or released by immune cells in a tumor environment (**Table 1**). For example, some miRNAs in EVs can inhibit gene expression and consequently alter interleukin levels. miR-23a-3p, miRNA present in EVs of hepatocellular carcinoma, are responsible for directing the inhibition of *PTEN* expression and increased phosphorylation of AKT and PD-L1 expression in macrophages, which leads to decreased IL-2 levels and CD8+ T cells in microenvironment (49). Cancer-derived EVs may also activate the tumor-associated macrophages and consequently activate the secretion of vascular endothelial growth factor (VEGF), IL-6, miRNAs, and transcription factors, which together promote angiogenesis, contributing to tumor progression. The participation of cancer-derived EVs with macrophages is one of the main pathways related to cytokine production in different tumor microenvironments (56, 57).

**Table 1:** Interleukins in cancer and their association with EVs.

Interleukin	Function	EV-secreting cell type	Effect of EVs on interleukins expression	Ref.
**IL-1**	Required for tumor invasion and angiogenesis	Melanoma cells	IL-1β upregulation	(48)
**IL-2**	Antitumoral	Hepatocellular carcinoma cells	Decreased	(49)
**IL-3**	Promotes hematological malignancies	CML cells	Drug delivery	(50)
**IL-6**	Protumoral: activates carcinogenesis	Colorectal cancer/melanoma cells	Upregulation	(34)
**IL-8**	Protumoral	CML, prostate cancer cells	Upregulation	(51, 52)
**IL-10**	Promotes cytotoxicity	Colorectal cancer cells	Upregulation	(34)
**IL-12**	Antitumoral	Melanoma/Colorectal cancer cells	Upregulation	(48, 53)
**IL-13**	Protumoral	Glioblastoma cells	Detection system IL-13 to quantum dots	(54)
**IL-23**	Protumoral	Colorectal cancer cells	Upregulation	(34)
**IL-26**	Protumoral *via* TH17 cells	Pancreatic cancer cells	Inhibition by miR-3607	(55)

For instance, in colorectal cancer (CRC), a study showed that SW620 (CRC cell line)-derived EVs could induce the secretion of IL-6, CXCL10, IL-23, and IL-10 in M0 macrophages (34). These results suggest that CRC cell line-derived EVs may reprogram immune cells’ immunophenotype and secretory profile (34). From this scenario of cell reprogramming conducted by EVs, cytokines such as IL-6 can induce phosphorylation events of transcriptional factors such as *STAT3*, contributing to tumor growth and metastasis of breast cancer (58). In melanoma, EVs can induce upregulation of markers to macrophages’ M1 and M2 polarization phenotypes such as CCL22, IL-12B, IL-1β, IL-6, i-NOS, and TNFα, promoting a protumor environment (48). Furthermore, EVs can induce angiogenic phenotypes, which can activate signal transduction in endothelial cells and thus lead to the release of IL-8 in the extracellular environment and the induction of an angiogenic phenotype (4). Therefore, the EVS can mediate key interactions in the microenvironmental tumor acting on immune cells by activating interleukin-related mechanisms in cancer.

Similarly, our group found that EVs released from the primary culture of prostatic tumor cells may promote the secretion of IL-8 in the extracellular environment (23). This event may contribute to the malignant transformation of non-tumor cells *in vitro* (23). Moreover, leukemia-derived exosomes may also active the IL-8 production in bone marrow stromal cells, protecting acute myeloid leukemia cells from apoptotic events resulting from chemotherapeutic treatments (51).

### EV-Associated Interleukins as a Diagnostic Tool

For many types of tumors, diagnosis can be invasive and uncomfortable process for the patient. Furthermore, late diagnosis can compromise the response to antitumor therapies. However, studies with EVs have shown that liquid biopsy can accelerate and improve approaches to diagnostic for many cancer types. Indeed, the evaluation of molecules such as mRNA, dsDNA, miRNA, protein, or cytokines isolated from EVs has been used in studies to detect cancer or correlation with metastatic events (59, 60). In pancreatic cancer, the serum levels of exosome miR-17-5p is higher in patients with this neoplasm than in healthy participants, suggesting the use of this EV-derived miRNA as a potential biomarker for pancreatic cancer (61). On the other hand, nanotechnology tools were used to demonstrated that the use of EVs-associated interleukins can be easily combined and coupled to nanoparticles in a tumor cell detection system (54). Thus, the authors reported that from the expression of the IL-13 receptor in EVs derived from glioblastoma, it was possible to build a detection system for this tumor by connecting IL-13 to quantum dots (IL13QD) (54). This complex can serve as a marker for glioma stem cells, and exosomes can inform the diagnosis and prognosis of patients with malignant disease. This interaction between EVs and cytokines enables the development of novel detection methods for cancer and the understanding of mechanisms associated with the tumor microenvironment. For instance, a recently study have showed that cytokines present in the tumor microenvironment could be conjugated to tumor-derived EVs and used to determine the uptake and biodistribution of EVs by cells expressing the cytokine receptor (62). In this study, the researchers also report that this conjunction between cytokines and EVs may be crucial for tumor promotion, altering the immune landscape (54, 62).

### EVs-Associated Interleukins as a Therapeutic Tool

**Figure 1:**
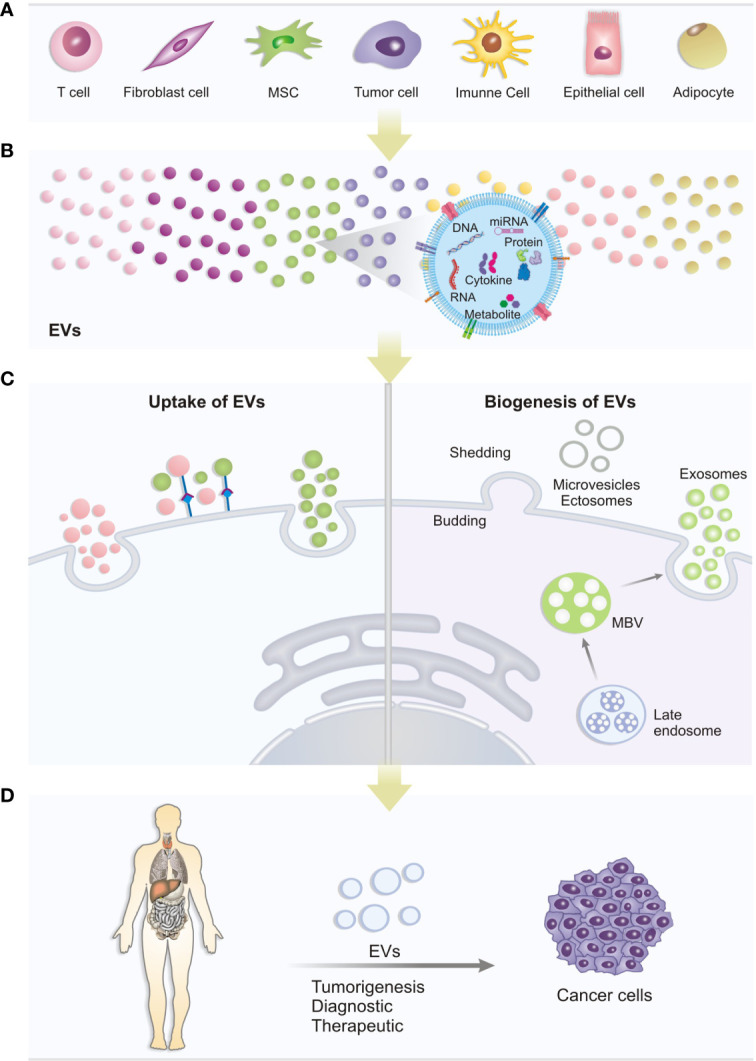
Biogenesis, uptake and role of EVs: **(A)** Different cell types can release different types of EVs under normal physiological or pathological conditions. **(B)** The EVs produced may contain several important molecules for cell communication, such as DNA, miRNA, Cytokines, proteins and metabolites that can trigger different biological responses in recipient cells. **(C)** Uptake of EVs: the mechanisms of extracellular vesicles-mediated transfer may be *via* target receptors present in the cytoplasmic membrane or direct fusion with the membrane. Interaction with membrane receptors can activate signaling pathways triggering cellular changes. Likewise, EVs, when fusing with the membrane, can release its content and generate cellular changes. Biogenesis: EVs can be produced by cells in different ways: Multivesicular bodies can develop from early endosomes and fuse with the plasma membrane releasing vesicles (exosomes) or, EVs can form by budding from the outer plasma membrane of the cell, with release of microvesicles to the extracellular environment. **(D)** In studies on the association of EVs with cancer, the potential of content carried by EVs to be used as a tool for new diagnostic strategies and therapeutic targets has been investigated.

The cargo of EVs, released in the cytoplasm of the target cell is presented as a potential source for discovering biomarkers, including cytokines and proteins. In addition, the association of EVs with cytokines, mediated or not by receptors, represents an exciting communication pathway between tumor and non-tumor cells that may be important for the development of therapeutic strategies (**Figure 1**). The therapeutic potential of EVs is significant in precision medicine for drug delivery (63, 64). Thus, despite the known role of EVs in tumorigenesis, some approaches have been explored to use these vesicles for therapeutic purposes. EVs fused with interleukin have been promising approaches for drug delivery systems and acquire antitumor potential as demonstrated in a study that evaluated the overexpression of the IL-3 receptor (IL3R), in chronic myeloid leukemia (CML) through the genetically engineering of HEK293T cells to produce IL-3 fused EVs. With that in mind, a delivery system was developed for the drug Imatinib for leukemic cells that overexpressed IL3R. The results obtained suggest that this Imatinib delivery system could be a novel tool for the treatment of CML since there was inhibiting tumor growth *in vivo* and *in vitro* (50). A study involving genetic modification of cell lines also showed potential for inhibiting tumor growth. In this research, MC38 cells were modified to overexpress IL-12, a proinflammatory interleukin, which was also overexpressed in EVs derived from these engineered cells. Thus, these EVs were used *in vivo* and *in vitro* studies to assess the antitumor immune response of this cytokine delivered *via* EVs (53). Similarly, another work showed that EVs released by renal cancer cells modified to express GPI-IL-12 (glycolipid-anchored-IL-12) could incorporate GPI-IL-12. This EV modification induces cytotoxic T lymphocytes, resulting in significant cytotoxic effects *in vitro* (65). These data suggest that EVs may have potential application in immunotherapy, emphasizing the production of vaccines based on EVs conjugated to antitumor interleukins for the treatment of renal cell carcinoma. In addition to this approach, EVs derived from immune cells can also inhibit tumor growth. A promising investigation showed that miR-3607-3p presents in EVs derived from NK cells can inhibit the malignant transformation and suppresses proliferation, migration, and invasion of pancreatic tumor cells, probably through the target IL-26 (55).

## Conclusion

Accumulated evidence suggests that EVs have immunotherapeutic potential, and their specific association with interleukins has enabled new approaches to the diagnosis of different types of cancer. Furthermore, with the advancement of personalized medicine, liquid biopsy has gained a prominent place in the clinical scenario with the use of genetically modified EVs as drug carriers, due to their non-toxic and non-immunogenic nature. However, despite the important studies presented here, the biological aspects that coordinate the migratory itinerary and the uptake of EVs by recipient cells, as well as the impact of genetic modification of EVs for therapeutic purposes, are still not fully understood. In addition, for an effective approach to EVs in clinical practice, large-scale production of these vesicles is necessary, as current isolation methods have low yields. In this sense, new efforts are needed to develop protocols that allow obtaining better yields of EVs isolated from biological fluids, as well as studies that make it possible to understand the biology of EVs both in the tumor microenvironment and their interaction with the host’s immune system. Altogether, future studies will be necessary to overcome those limitations before applying EVs in clinical practice.

## Author Contributions

AS performed the literature search and writing. LC contributed to draft preparation, writing, and review. All authors contributed to the article and approved the submitted version.

## Funding

This work was funded by Sao Paulo State Research Council (FAPESP), grant number 2020-10960-5.

## Conflict of Interest

The authors declare that the research was conducted in the absence of any commercial or financial relationships that could be construed as a potential conflict of interest.

The handling Editor FC declared a shared affiliation with the authors.

## Publisher’s Note

All claims expressed in this article are solely those of the authors and do not necessarily represent those of their affiliated organizations, or those of the publisher, the editors and the reviewers. Any product that may be evaluated in this article, or claim that may be made by its manufacturer, is not guaranteed or endorsed by the publisher.
